# The effect of reminder mobile application use on medication adherence after total thyroidectomy: a randomized controlled trial

**DOI:** 10.1007/s00520-026-10533-0

**Published:** 2026-03-09

**Authors:** Pınar Kaya, Sevilay Erden

**Affiliations:** 1 Department of Medical Services and Techniques, Eğirdir Health Services Vocational School, Süleyman Demirel University, Isparta, Turkey; 2 Faculty of Health Sciences, Department of Nursing, Çukurova University, Adana, Turkey

**Keywords:** Health education, Reminder systems, Levothyroxine, Perioperative nursing, Medication adherence

## Abstract

**Purpose:**

This study aimed to investigate the effect of using a reminder-based mobile application on medication adherence in patients who underwent total thyroidectomy.

**Materials and methods:**

This randomized controlled trial followed CONSORT guidelines and included 63 patients who underwent total thyroidectomy at a university and a state hospital. The intervention group (*n* = 31) used a reminder mobile application for 8 weeks, while the control group (*n* = 32) received standard care. Data were collected at weeks 1, 4, and 8 using a personal information form, follow-up form, and Morisky eight-item medication adherence scale. Statistical analyses included chi-square, *t*-test, ANOVA, Mann–Whitney *U*, and Friedman tests.

**Results:**

At week 4, the intervention group demonstrated significantly higher mean MMAS-8 scores compared to the control group (6.58 vs. 5.71, *p* = 0.016). Furthermore, at week 8, a statistically significant difference was observed between groups in terms of medication adherence levels based on MMAS-8 cutoff categories (*χ*^2^ = 9.6, *p* = 0.008).

**Discussion:**

This study showed that a reminder-based mobile application effectively improved medication adherence in patients after total thyroidectomy. Increased adherence was observed in the intervention group, while a decline occurred in the control group. These findings indicate that reminder-based mobile health interventions may support medication adherence during the early postoperative period.

**Conclusion:**

Reminder mobile applications can positively impact medication adherence. Integrating such tools into routine postoperative follow-up may support treatment continuity in patients receiving levothyroxine after total thyroidectomy.

**ClinicalTrials.gov identifier:**

NCT05503576, registration date 08 December 2022.

**Supplementary Information:**

The online version contains supplementary material available at 10.1007/s00520-026-10533-0.

## Introduction

Total thyroidectomy is a commonly performed surgical procedure for the treatment of multinodular goiter, Graves’ disease, toxic multinodular goiter, and thyroid malignancies. As the entire thyroid gland is removed in total thyroidectomy, levothyroxine replacement therapy is administered to compensate for hormone deficiency and to prevent postoperative complications [1, 2]. When the optimal dose of levothyroxine is properly adjusted and maintained, side effects occur at a very low rate [3]. Following initiation of levothyroxine therapy after total thyroidectomy, TSH levels are typically evaluated at 6–8 weeks, highlighting the importance of assessing early adherence behavior before any clinical dose modifications. Once the optimal dose is reached, TSH monitoring is recommended every 4–6 months initially, and subsequently once a year [4, 5]. Levothyroxine is considered a narrow therapeutic index (NTI) drug, in which small variations in dosage or bioavailability may lead to significant alterations in serum thyroid hormone concentrations [6]. This pharmacological characteristic makes consistent medication adherence particularly critical.

Although the appropriate dose is determined over time, thyroid hormone levels may become unstable in the long term. Factors contributing to this instability include comorbid conditions, changes in body mass index and dietary habits, and poor medication adherence [7]. Patients may face lifelong risks such as hypocalcaemia, hypothyroidism, or hyperthyroidism following total thyroidectomy [8, 9]. Therefore, it is essential to educate patients on complication management and to promote consistent medication adherence.

Medication errors are frequently reported after total thyroidectomy [1, 2, 10]. Maintaining medication adherence after total thyroidectomy requires permanent lifestyle adjustments. El Helou et al. (2019) reported that only 14.5% of patients taking levothyroxine demonstrated high adherence; however, adherence was evaluated at a mean hypothyroidism duration of 9.71 ± 7.52 years, not at a defined time point following thyroidectomy [1]. Research has shown that improving medication adherence may have a more significant impact on patient outcomes than developing new medical treatments [11]. Factors such as regular medication use, monitoring of serum drug levels, and access to up-to-date and reliable health information in the event of dosing errors can enhance adherence after discharge [12].

The most widely recommended method for measuring medication adherence is the use of standardized and validated self-report tools. Among these, the Morisky eight-item medication adherence scale (MMAS-8) is the most frequently employed in the literature [10, 13].

The World Health Organization (WHO) estimates that more than 50% of patients do not adhere to their prescribed medication regimens [14]. Similar findings have been reported in other studies evaluating medication adherence among patients requiring long-term pharmacological treatment [15]. To date, various mobile health (mHealth) interventions have demonstrated effectiveness in enhancing medication adherence [12]. However, the number of studies specifically investigating the impact of mobile applications on adherence in the early postoperative period remains limited [12, 16]. Given that patients undergoing total thyroidectomy require lifelong levothyroxine therapy postoperatively, medication non-adherence represents a significant concern in this population. A review of the literature revealed no studies assessing the impact of reminder-based mobile applications on medication adherence following total thyroidectomy. Accordingly, this randomized controlled trial aimed to evaluate medication adherence as a behavioral outcome regarding the use of a mobile reminder application among patients receiving levothyroxine replacement therapy in the early postoperative period after total thyroidectomy. Specifically, medication adherence levels of patients using a mobile reminder application were compared with those receiving standard care over an 8-week follow-up period. The subsequent sections of the manuscript present the study design and methodology, followed by the results of the intervention, a discussion of the findings in relation to existing literature, and conclusions regarding the implications for postoperative nursing care.

## Method

### Research design

This study is a randomized controlled interventional trial designed in accordance with the Consolidated Standards of Reporting Trials (CONSORT) guidelines.

The sample consisted of patients who underwent total thyroidectomy in the general surgery departments of two hospitals in Turkey—one a university hospital and the other a state hospital. Eligible participants were between 18 and 70 years of age, had undergone total thyroidectomy due to benign thyroid disease, had no diagnosed mental disorders, were able to speak Turkish, and were capable of using an Android-based mobile device.

Eligible participants were identified during their preoperative anesthesia clinic visit. After providing consent, patients were randomized preoperatively into either the intervention or control group. Block randomization was used to ensure balanced group sizes and minimize selection bias. The random allocation sequence was generated using the Randomizer.org website and implemented by an independent faculty member who was not involved in the study procedures.

**Fig. 1:**
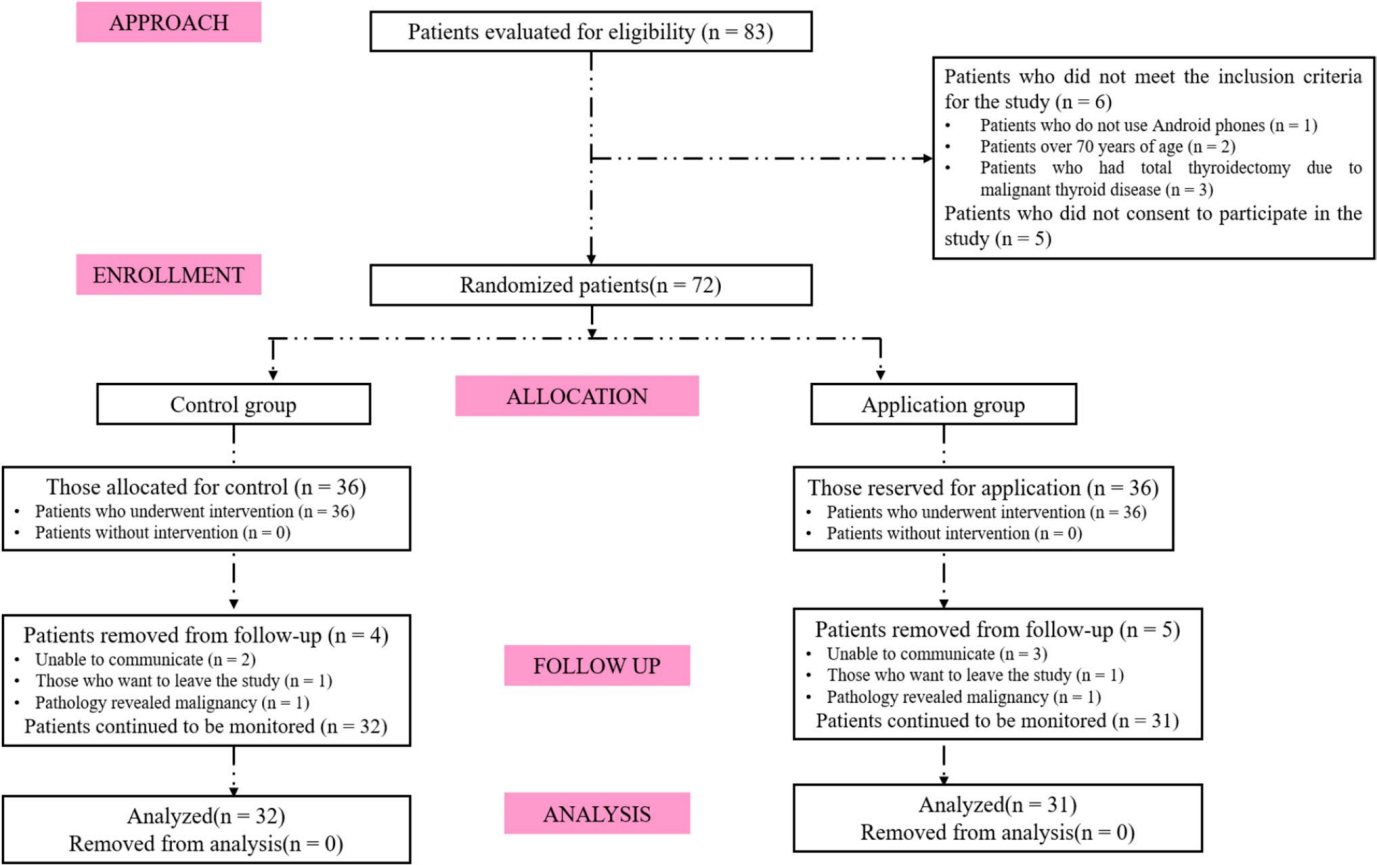
CONSORT 2018 flowchart

The sample size was determined based on a power analysis using preliminary data from 20 patients (10 in the control group and 10 in the intervention group). The power analysis was conducted using the G*Power 3.1.9.2 software (Universität Kiel, Germany). Assuming a two-tailed test, 90% power, a 0.05 alpha level, and an effect size of *d* = 0.426, the required minimum sample size was calculated to be 60 participants (*n*₁ = 30, *n*₂ = 30). To account for potential participant dropout during data collection, the sample was increased by 20% as recommended in the literature [17]. Ultimately, the study sample consisted of 63 patients (32 in the control group and 31 in the intervention group) (Fig. 1).

### Data collection tools

The data for this study were collected using a personal information form, a follow-up form, and the MMAS-8, all developed or selected based on relevant literature.

- *Personal information form:* This form was developed by the researcher following a comprehensive literature review and includes ten items designed to collect descriptive data about the patient. Variables include age, sex, weight, height, marital status, presence of chronic diseases, employment status, education level, household income, and family structure [1, 10, 11, 18] The form is provided in Online Resource 1.
- *Follow-up form:* Also developed by the researcher based on the literature, this form consists of three items that record the name and dosage of the medication used by the patient and whether any complications developed [19]. The form is provided in Online Resource 2.
- *MMAS-8:* Originally developed by Morisky et al. (1986) to assess medication adherence among patients with hypertension, the scale initially consisted of four items [20]. The case management adherence guideline later introduced two additional items, resulting in the current modified version [21]. The MMAS-8 is a self-reported measure that evaluates patients’ medication-taking behavior. The Turkish version of the scale was validated and its reliability confirmed by Oğuzülgen et al. in 2014. The scale comprises eight items. The first seven questions are answered with “Yes” or “No.” Items 1–4, 6, and 7 are scored as 1 point for “No” and 0 points for “Yes”; item 5 is scored inversely, with 1 point for “Yes” and 0 points for “No.” Item 8 is a 5-point Likert-type question, where only the “Never” response scores 1 point; all other responses score 0. Higher total scores indicate greater adherence (low adherence, < 6 points; medium adherence, 6–7 points; high adherence, 8 points) [22].
- *Reminder mobile application:* The mobile application used in this study was developed to improve medication adherence among patients who had undergone total thyroidectomy through a structured, multi-stage process including content development, expert evaluation, software implementation, usability testing, and final revision.

Educational content and reminder modules were prepared based on current literature on levothyroxine use and postoperative management. The material was reviewed by a multidisciplinary expert panel using the DISCERN instrument, demonstrating high reliability and quality with strong inter-rater agreement.

The application interface was designed using Android Studio and coded in Java, with Firebase employed for secure data storage, synchronization, and centralized management. The system included patient and administrator roles to enable controlled access and monitoring of entries.

Usability testing was conducted with five patients using the System Usability Scale, yielding high usability scores. Feedback from this phase informed final refinements, and the application was subsequently implemented in the intervention phase. The application provided educational content and medication reminders to support adherence during the postoperative period. To enhance transparency and reproducibility, representative screenshots of the mobile application used in the intervention group are presented in Online Resource 3.

### Intervention and control groups

Patients were approached and informed about the study during the preoperative period, and written informed consent was obtained before surgery. Postoperative follow-up measurements were initiated in the first week after total thyroidectomy for both groups.

All participants were prescribed levothyroxine in tablet (solid) form as part of standard postoperative management. Although individual doses were calculated according to clinical evaluation, the pharmaceutical formulation was identical for all patients, ensuring comparability between the intervention and control groups. Therefore, potential differences in medication adherence related to levothyroxine formulation were minimized and could not have confounded the comparison between groups. No levothyroxine dose adjustments were made during this period in accordance with standard postoperative treatment protocols.

Following group allocation, patients in the intervention group were introduced to the mobile application using an Android device on which the application had already been installed. Subsequently, with assistance from the researcher, the application was downloaded and set up on the patient’s personal Android smartphone using the hospital’s free internet service. A unique username and password for each patient were generated by the researcher through the administrator panel of the application. The orientation and setup process took approximately 20 min per patient.

**Fig. 2:**
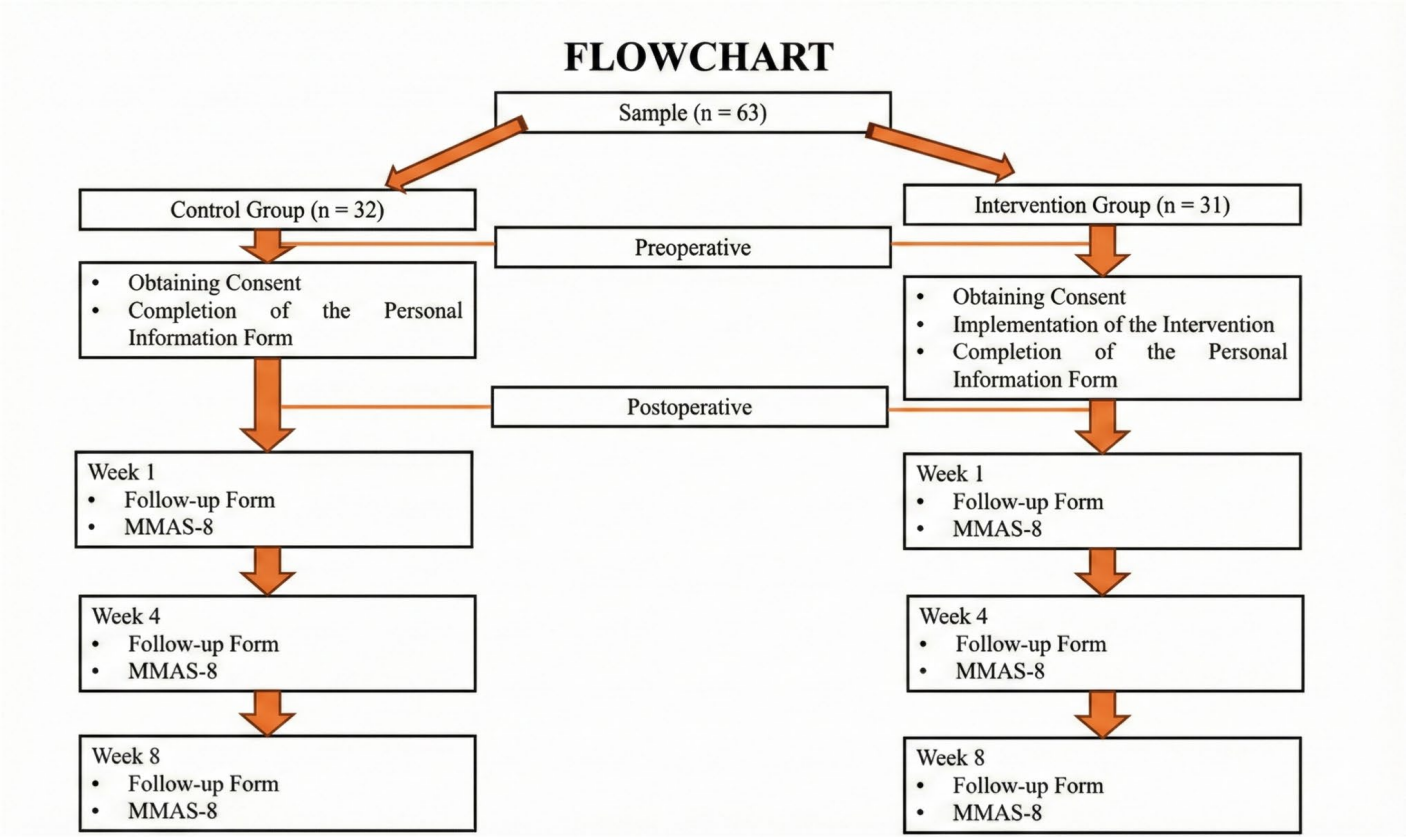
Flowchart of the study

Patients were informed that the follow-up form and MMAS-8 within the application were to be completed during the 1 st, 4th, and 8th weeks following their enrolment (Fig. 2).

In the control group, following patient group assignment and the initial meeting where informed consent was obtained, participants were asked to complete the personal information form. In the subsequent 1 st, 4th, and 8th weeks, a link containing the follow-up form and the *MMAS-8* was sent to the patients via SMS. Participants were instructed to complete the data collection forms using Google Forms.

### Data analysis

The research data were analyzed using the Statistical Package for the Social Sciences (SPSS) version 20. Descriptive statistics, including frequency, percentage, mean, standard deviation, minimum, maximum, and median, were used to summarize the data. For variables showing normal distribution, independent-samples *t*-test and repeated-measures ANOVA were employed. Chi-square (*χ*^2^) tests and cross-tabulations were used to examine the relationships between categorical variables. The normality of the data was assessed by examining skewness and kurtosis values as well as Q-Q plots, and the results confirmed that the data met the assumptions of normal distribution. Accordingly, parametric methods were used in the analysis. Bonferroni correction was applied for multiple comparisons of main effects. Effect size was calculated using eta-squared (*η*^2^) coefficients, which indicate the practical significance of differences between groups. Eta-squared values were interpreted as follows: 0.01 = small, 0.06 = medium, and 0.14 = large effect sizes [23]. For all statistical analyses, a significance level of *p* < 0.05 was considered statistically significant.

The primary outcome of the study was medication adherence measured using the MMAS-8, reflecting behavioral adherence during the early postoperative period. Biochemical thyroid function parameters were not included as outcome variables. The study hypotheses were defined as follows:

- H₀: There is no statistically significant difference in the mean total scores of the MMAS-8 between the patient group using the mobile application and the control group.
- H₁: There is a statistically significant difference in the mean total scores of the MMAS-8 between the patient group using the mobile application and the control group.

### Ethical considerations

Approval for the study was obtained from the Non-Interventional Clinical Research Ethics Committee of the University (Meeting No: 121, Decision No: 83). Written permission was also granted by the Provincial Health Directorate, and authorization to use the relevant scales in the study was obtained via email correspondence with the original authors. Informed consent, both verbal and written, was obtained from all participants after they were informed about the study.

The study was conducted in accordance with the principles of the Declaration of Helsinki and adhered to ethical standards for research and publication. Additionally, a flow diagram was prepared in line with the CONSORT 2018 guidelines (Fig. 1).

## Results

There were no statistically significant differences between the intervention and control groups in terms of demographic and clinical characteristics (*p* > 0.05) (Table 1 and Table 2). A statistically significant difference was found between the intervention and control groups in terms of medication adherence levels at week 8 (*χ*^2^ = 9.6, *p* = 0.008) (Table 3). When examining within-group MMAS-8 total score means, the control group showed significantly higher scores in week 1 compared to weeks 4 and 8. The interaction between time and the control group was found to be statistically significant with a large effect size (*F* = 11.72, *p* < 0.001, *η*^2^ = 0.268).

**Table 1:** Comparison of demographic characteristics between groups

Variables	Control group (***n*** = 32)		Intervention group (***n*** = 31)		Test
	*n*	%	*n*	%	
Gender					*χ*^2^ = 0.141^a^
Female	26	81.3	24	77.4	*p* = 0.707
Male	6	18.8	7	22.6	
Marital status					*χ*^2^ = 0.454^a^ *p* = 0.501
Single	6	18.8	8	25.8	
Married	26	81.3	23	74.2	
Chronic disease					*χ*^2^ = 0.273^a^ *p* = 0.098
No	18	56.3	11	35.5	
Yes	14	43.8	20	64.5	
Employment status					*χ*^2^ = 0.176^a^ *p* = 0.674
Unemployed	19	59.4	20	64.5	
Employed	13	40.6	11	35.5	
Education level					*χ*^2^ = 3.97^a^ *p* = 0.410
Literate	2	6.3	5	16.1	
Primary education	12	37.5	10	32.3	
Secondary education	12	37.5	9	29	
Higher education	6	18.8	5	16.1	
Graduate education	0	0	2	6.5	
Family type					*χ*^2^ = 1.07^a^ *p* = 0.300
Nuclear family	21	65.6	24	77.4	
Extended family	11	34.4	7	22.6	
Income status					*χ*^2^ = 4.90^a^ *p* = 0.086
Income less than expenses	14	43.8	19	61.3	
Income equal to expenses	14	43.8	12	38.7	
Income greater than expenses	4	12.5	0	0	
	***x̄*** ± SD	Median (Min–Max)	***x̄*** ± SD	Median (Min–Max)	
Age (years)	48.6 ± 7.9	48 (32–68)	51.1 ± 10.5	52 (31–68)	*t *= −1.054^b^ *p* = 0.296
Height (centimeters)	163.2 ± 10.7	165 (145–186)	166.6 ± 12,2	165 (148–189)	*t* = −1.198^b^ *p* = 0.236
Weight (kilograms)	79.2 ± 14.1	77 (59–118)	76.1 ± 16.1	73 (50–108)	*t* = −0.811^b^ *p* = 0.421

*SD*, standard deviation

^a^Pearson-*χ*^2^

^b^Independent-samples *t*-test

**Table 2:** Comparison of follow-up characteristics between the groups

Variables	Control group (***n*** = 32)		Intervention group (***n*** = 31)		Test
	*n*	%	*n*	%	
Occurrence of complications					*χ*^2^ = 0.043^a^ *p* = 0.836
No	23	71.9	23	74.2	
Yes	9	28.1	8	25.8	
Knowledge of medication name (week 1)					*χ*^2^ = 1.49^a^ *p* = 0.222
Does not know	8	25	4	12.9	
Knows	24	75	27	87.1	
Knowledge of medication name (week 4)					*χ*^2^ = 2.15^a^ *p* = 0.143
Does not know	6	18.8	2	22.6	
Knows	26	81.3	29	77.4	
Knowledge of medication name (week 8)					*χ*^2^ = 0.318^a^ *p* = 0.573
Does not know	2	6.3	1	3.2	
Knows	30	93.8	30	96.8	
Knowledge of medication dosage (week 1)					*χ*^2^ = 0.38^a^ *p* = 0.535
Does not know	22	68.8	19	61.3	
Knows	10	31.3	12	38.7	
Knowledge of medication dosage (week 4)					*χ*^2^ = 0.01^a^ *p* = 0.930
Does not know	10	31.3	10	32.3	
Knows	22	68.8	21	68.3	
Knowledge of medication dosage (week 8)					*χ*^2^ = 0.23^a^ *p* = 0.633
Does not know	10	31.3	8	25.8	
Knows	22	68.8	23	74.2	

^a^Pearson *χ*^2^

**Table 3:** Comparison of medication adherence between groups according to measurement time points

MMAS-8 medication adherence scale	Control group (*n* = 32)		Intervention group (*n* = 31)		Test
	*n*	%	*n*	%	
Medication adherence level at week 1					*χ*^2^ = 1.49^a^ *p* = 0.476
Low	8	25	11	35.5	
Medium	18	56.3	17	54.8	
High	6	18.8	3	9.7	
Medication adherence level at week 4					*χ*^2^ = 3.65^a^ *p* = 0.161
Low	13	40.6	6	19.4	
Medium	15	46.9	18	58.1	
High	4	12.5	7	22.6	
Medication adherence level at week 8					*χ*^2^ = 9.60^a^ *p* = 0.008*
Low	16	50	10	32.3	
Medium	14	43.8	9	29	
High	2	6.3	12	38.7	
Medication adherence measurement time points	Mean	SD	Mean	SD	Test
Week 1	6.34	1.31	5.61	1.66	*t*^b^ = 1.938 *p* = 0.057
Week 4	5.71	1.59	6.58	1.11	*t*^b^ = −2.480* *p* = 0.016*
Week 8	5.28	1.59	6.16	2.19	*t*^b^ = −1.828 *p* = 0.074
***F***^***c***^	11.372		4.264		
***P***	0.001*		0.019*		
Bonferroni	1 > 2.3*		1 < 2*		
*η*^2^	0.268		0.124		

*η*^*2*^  eta squared, *SD* standard deviation

^b^Independent-samples *t*-test

^c^Repeated-measures ANOVA

**Fig. 3:**
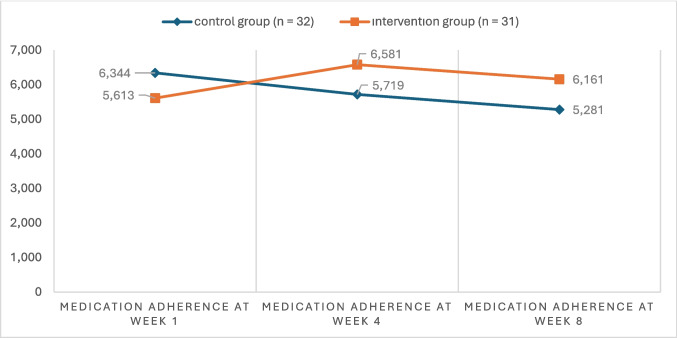
Distribution of medication adherence across measurement time points by group

**Fig. 4:**
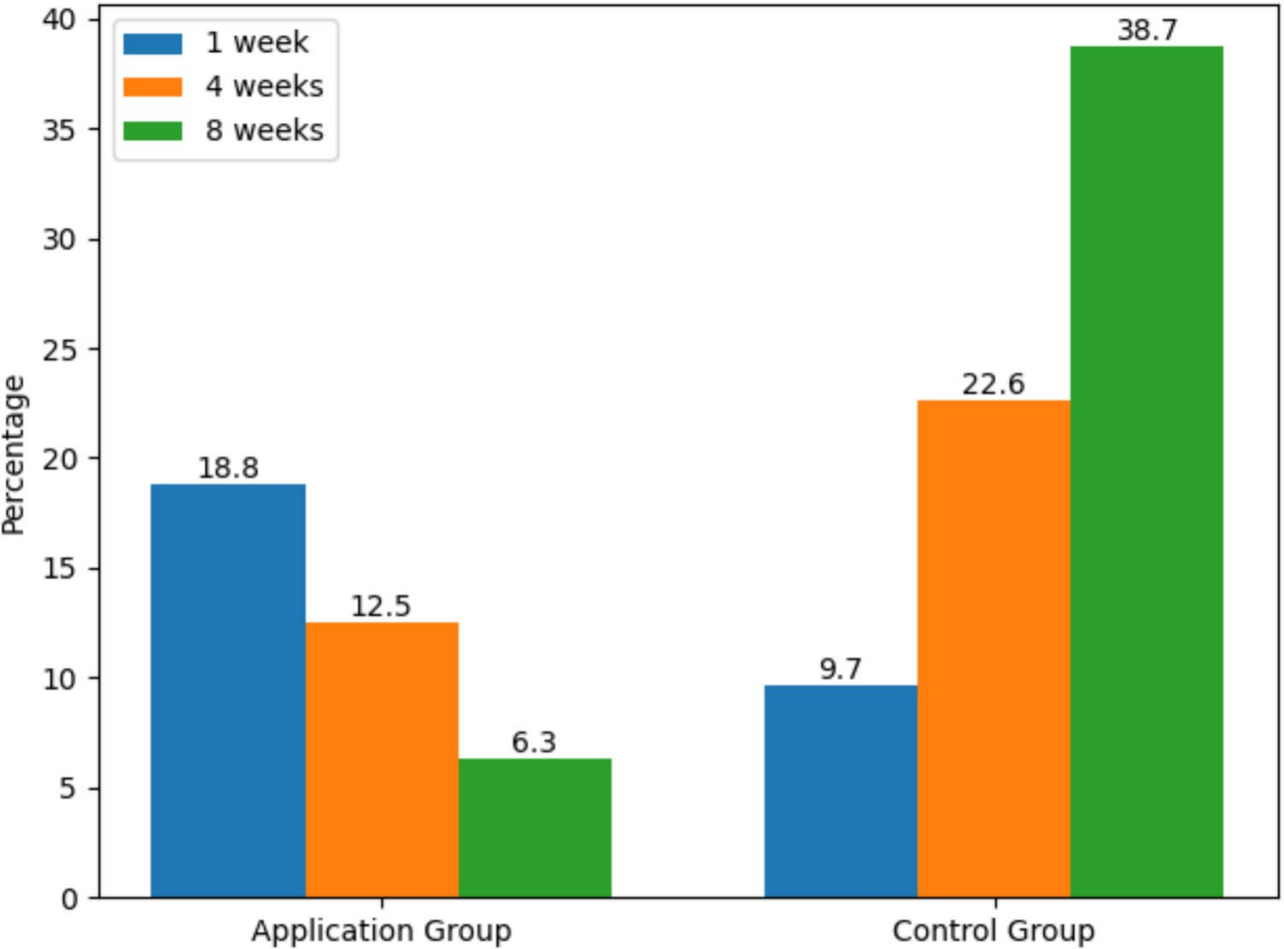
Changes in the rates of high medication adherence over measurement time points by group

In the intervention group, the MMAS-8 total score in week 4 was statistically significantly higher than the score in week 1. The time*group interaction was also significant with a moderate effect size (*F* = 4.264, *p* = 0.019, *η*^2^ = 0.124) (Table 3). While the control group’s medication adherence scores declined over time, the intervention group’s scores showed an increase compared to baseline. The mean scores in weeks 4 and 8 were higher in the intervention group than in the control group (Figs. 3 and 4). When the changes in high medication adherence levels were examined across different measurement times, a decrease was observed in the control group, whereas an increase was noted in the intervention group (Figs. 3 and 4).

## Discussion

Levothyroxine replacement therapy is required as a chronic treatment to maintain euthyroidism after total thyroidectomy [9, 24–27]. The effectiveness of levothyroxine therapy is directly related to medication adherence [28]. Various studies in the literature have examined the effects of different approaches on medication adherence [13, 19, 29–31]. Among these approaches, mobile applications have recently become a new trend due to factors such as increasing patient-nurse interaction and ease of access [13, 16, 30, 32]. However, there is limited evidence regarding the effect of mobile applications on medication adherence [12, 16]. Medication non-adherence is a significant issue for total thyroidectomy patients [18, 28, 33–35]. To our knowledge, no studies have investigated the impact of reminder mobile applications on medication adherence in total thyroidectomy patients. Therefore, our findings were discussed in light of other studies on levothyroxine adherence and postoperative medication adherence.

In our study, in the control group, 18.8% of patients had high, 56.3% moderate, and 25% low medication adherence in the 1 st week. In the application group, 9.7% had high, 54.8% moderate, and 35.5% low adherence. Most adherence studies have assessed patients in stable, long-term treatment phases rather than within the first postoperative week; therefore, direct comparison of week 1 adherence distributions is limited [1, 10, 18, 36]. No statistically significant difference was observed between the groups during the 1 st week. This finding may reflect the unique characteristics of the early postoperative period, when medication use may be partially supported by caregivers and family members in the immediate postoperative period, a factor known to influence adherence behaviors, potentially leading to more standardized medication-taking patterns across patients regardless of intervention[36].

Karataş and Hacıoğlu (2022) found that 57.4% of hypothyroid patients had moderate/high medication adherence, and treatment success was 55.6%. They concluded that treatment success was positively correlated with levothyroxine adherence [28]. As suggested in other studies, interventions to increase medication adherence are necessary to improve treatment success [11, 29, 30, 37, 38]. In our study, the proportion of patients showing moderate or high adherence increased at weeks 4 and 8 compared to week 1 in the mobile application group, indicating the effectiveness of the intervention.

Yu et al. (2020) conducted a multicenter randomized controlled trial assessing the effect of mobile application use on medication adherence after coronary artery bypass graft surgery and found no significant effect (*p* = 0.749). However, mobile app usage sharply declined over time (88.1% in month 1, 42.5% in month 2, and 9.2% in month 6) [19]. In our study, changes in adherence patterns over time may also be influenced by variations in motivation and engagement with the application; the decrease in adherence at week 8 in the application group might be explained by similar reductions in app usage or declining motivation over time [19, 39]. Factors such as reduced perceived necessity of treatment, forgetfulness, and absence of early complications may also contribute to decreased adherence [40]. Nonetheless, while adherence rates in the control group declined from the first week onward, they increased in the application group (Fig. 3).

Studies evaluating medication adherence among patients using levothyroxine have reported varying rates of high, moderate, and low adherence [1, 10, 18, 41]. However, these findings largely reflect patients receiving long-term therapy or those in routine follow-up, where medication-taking behaviors are already established. In contrast, our study assessed adherence during the early postoperative period following total thyroidectomy, when levothyroxine therapy is newly initiated and patients are still adapting to lifelong treatment routines. Therefore, differences in clinical context and timing of assessment should be considered when comparing adherence levels across studies.

Literature suggests that medication adherence typically decreases over time following the initial prescription [42, 43]. Consistent with this, the control group in our study showed a significant decrease in MMAS-8 total scores with a large effect size from the first week onward (*F* = 11.72, *p* < 0.001, *η*^2^ = 0.268). At week 4, the mean MMAS-8 scores in the application group were significantly higher than in the control group (*t* = −2.48, *p* = 0.016), suggesting the effectiveness of the reminder-based mobile application. Similarly, Santo et al. (2019) found that mobile application use improved medication adherence in patients with coronary artery disease over a 3-month follow-up (*p* = 0.008) [44].

Although the difference in mean MMAS-8 scores between groups at week 8 was not statistically significant, the application group (6.16 ± 2.19) scored higher than the control group (5.28 ± 1.59). Recalculation of the scale scores considering the Likert-type last item showed significantly higher scores for the application group at weeks 4 and 8 (*Z* = 313, *p* = 0.010; and *Z* = 344, *p* = 0.034). These results underscore the importance of considering the adapted scoring system in future research [22].

It is important to interpret these findings in relation to the timing of adherence assessment following treatment initiation. Medication adherence was first evaluated in the early postoperative period (week 1), when patients may still demonstrate high motivation and closer clinical supervision. The significant improvement observed at week 4 suggests that the mobile reminder application was particularly effective during the early adaptation phase of lifelong levothyroxine therapy. By week 8, adherence behaviors may begin to stabilize, and natural declines in motivation could influence outcomes. Therefore, the timing of adherence measurement should be considered when interpreting the effectiveness of the intervention.

In conclusion, our findings support the hypothesis that reminder mobile application use effectively promotes medication adherence. The application’s reminder and educational features appear beneficial for medication management. However, additional strategies are needed to sustain long-term engagement with mobile applications. In addition, further studies incorporating objective biochemical outcomes are needed to determine the clinical impact of enhanced adherence.

### Strengths and limitations

This study has several strengths. It was designed as a randomized controlled trial and conducted in accordance with CONSORT guidelines. The mobile application was systematically developed based on the literature, validated by expert review using the DISCERN instrument, and tested for usability prior to implementation. Additionally, the intervention was conducted in real clinical settings, increasing its practical relevance.

However, certain limitations should be acknowledged. The follow-up period was limited to 8 weeks, and longer-term adherence outcomes were not evaluated. Medication adherence was assessed using a self-reported measure, which may introduce reporting bias. Biochemical thyroid function parameters, such as TSH, were not evaluated as outcome measures; therefore, the direct clinical impact of improved adherence could not be objectively confirmed within the follow-up period. Furthermore, only patients using Android-based devices were included, which may restrict applicability to broader populations.

Future studies with larger and more diverse samples and longer follow-up periods are recommended to confirm and extend these findings.

### Practice implications

The findings of this study show that a mobile application-based intervention involving structured patient education significantly improves medication adherence in patients receiving levothyroxine treatment after total thyroidectomy. The educational modules included in the app provided patients with accessible and understandable information about their condition, the proper timing of medication use, and the potential consequences of non-adherence, thereby enhancing patients’ active participation in the process and strengthening their self-management skills. These results suggest that integrating mobile health tools enriched with patient education into the postoperative routine can improve treatment adherence, may contribute to improved treatment consistency, and reduce the burden on healthcare services. Healthcare professionals and institutions should consider evaluating such digital interventions to support patient-centered care approaches in thyroid hormone replacement therapy.

## Supplementary Information

Below is the link to the electronic supplementary material.

ESM 1 (DOCX.16.7 KB)

ESM 2 (DOCX.13.8 KB)

ESM 3 (DOCX.511 KB)

## Data Availability

No datasets were generated or analysed during the current study.
