# Supplementary material for: The effect of reminder mobile application use on medication adherence after total thyroidectomy: a randomized controlled trial
Source: Support Care Cancer. 2026 Mar 9;34(4):295. doi: 10.1007/s00520-026-10533-0 (PMC12968104; doi:10.1007/s00520-026-10533-0)
Supplement: Supplementary file 1 — (DOCX.16.7 KB) [file 520_2026_10533_MOESM1_ESM.docx]

Online Resource 1. Personal Information Form

| Age | **………….** |
| --- | --- |
| Sex | - Male - Female |
| Weight | …………..kg |
| Height | …………..cm |
| Marital status | - Single - Married |
| Presence of chronic disease | - No - Yes. Please specify (………………………………………) |
| Employment status | - No - Yes |
| Education level | - Literate - Primary education - Secondary education - Higher education - Graduate education |
| Income status | - Income less than expenses - Income equal to expenses - Income greater than expenses |
| Family type | - Nuclear family - Extended family |
