# Supplementary material for: The effect of reminder mobile application use on medication adherence after total thyroidectomy: a randomized controlled trial
Source: Support Care Cancer. 2026 Mar 9;34(4):295. doi: 10.1007/s00520-026-10533-0 (PMC12968104; doi:10.1007/s00520-026-10533-0)
Supplement: Supplementary file 2 — (DOCX.13.8 KB) [file 520_2026_10533_MOESM2_ESM.docx]

Online Resource 2. Follow-up Form

| Medication name: |  |
| --- | --- |
| Dosage: |  |
| Occurrence of complications: |  |
