# Supplementary material for: The effect of reminder mobile application use on medication adherence after total thyroidectomy: a randomized controlled trial
Source: Support Care Cancer. 2026 Mar 9;34(4):295. doi: 10.1007/s00520-026-10533-0 (PMC12968104; doi:10.1007/s00520-026-10533-0)
Supplement: Supplementary file 3 — (DOCX.511 KB) [file 520_2026_10533_MOESM3_ESM.docx]

**Online Resource 3. Screenshots of the mobile application used in the intervention group**

The images illustrate the main components of the application, including the login screen, home page, educational modules, follow-up form, and reminder interface. No real patient data were used in the screenshots. A simulated user account was created solely for demonstration and documentation of the application interface. These visuals are provided to improve transparency and reproducibility of the intervention.

| 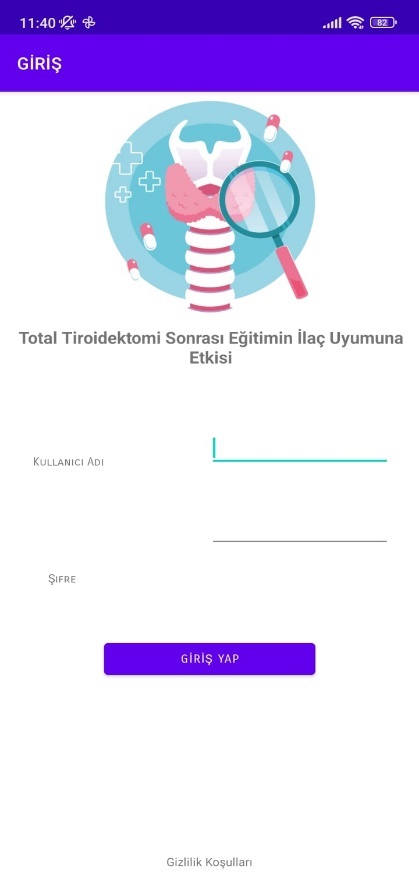 | 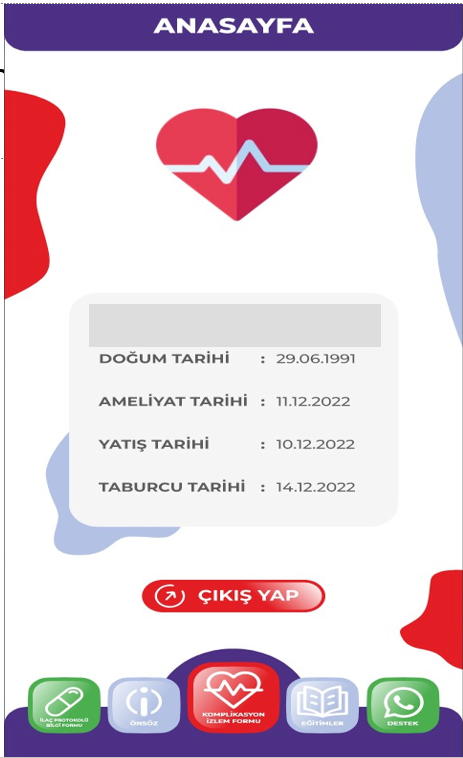 | 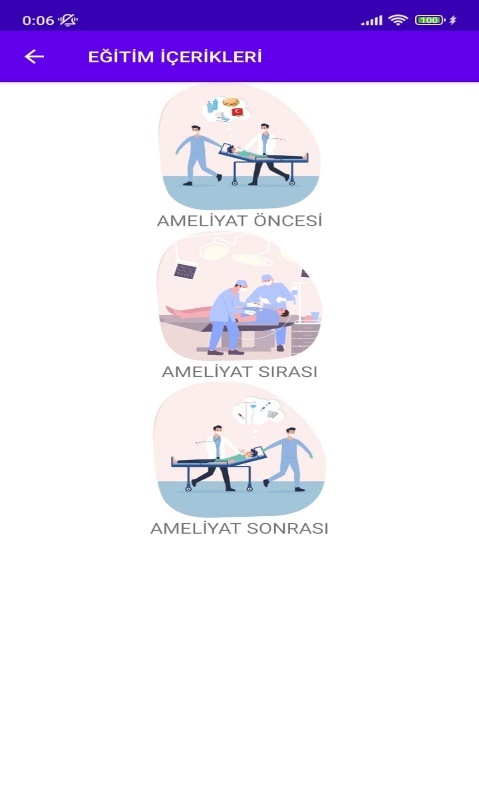 |
| --- | --- | --- |
| Figure S1. Login screen | Figure S2. Home page | Figure S3. Educational content |

| 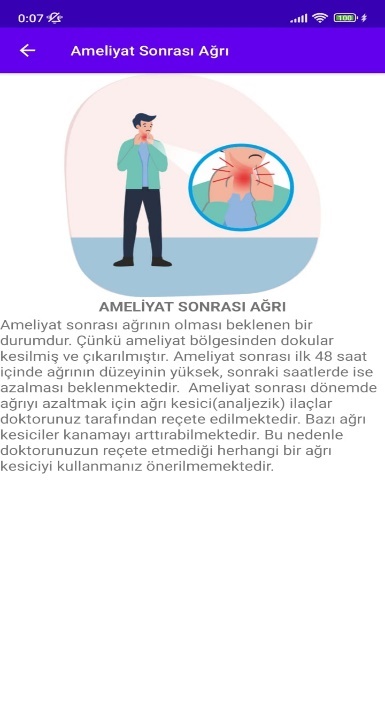 | 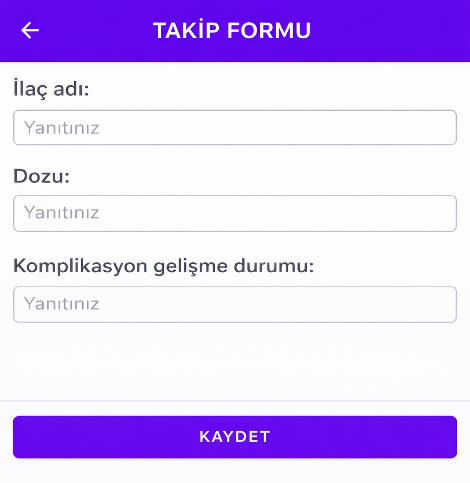 | 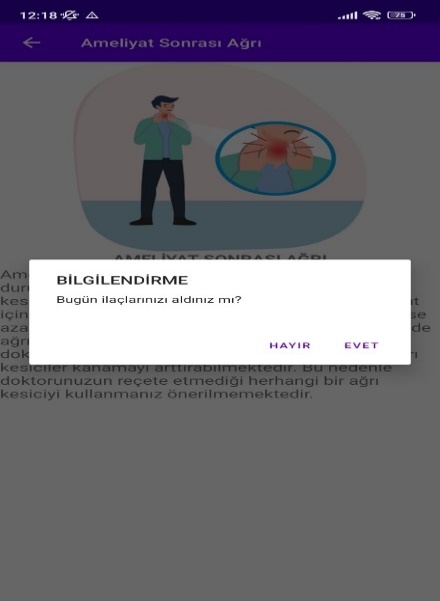 |
| --- | --- | --- |
| Figure S4. Education page | Figure S5. Follow-up form | Figure S6. Reminder screen |
